# Anthropogenic Infrastructures Shape Brown Bear Movements in Human‐Modified Landscapes

**DOI:** 10.1002/ece3.72680

**Published:** 2026-02-26

**Authors:** Pino García‐Sánchez, Vincenzo Penteriani, María del Mar Delgado, Daniele Falcinelli, Ancuta Fedorca, Louise K. Gentle, Ilpo Kojola, Samuli Heikkinen, Slavomír Find'o, Michaela Skuban, Mihai Fedorca, Ovidiu Ionescu, Georgeta Ionescu, Ramon Jurj, Marius Popa, Andrés Ordiz, Jon E. Swenson, Antonio Uzal

**Affiliations:** ^1^ School of Animal, Rural and Environmental Sciences Nottingham Trent University, Brackenhurst Campus Nottinghamshire UK; ^2^ Department of Wildlife National Institute for Research and Development in Forestry Marin Dracea Brasov Romania; ^3^ Department of Evolutionary Ecology National Museum of Natural Sciences (MNCN‐CSIC) Madrid Spain; ^4^ Biodiversity Research Institute (IMIB, CSIC‐Oviedo University‐Principality of Asturias) Mieres Spain; ^5^ Department of Biology and Biotechnologies “Charles Darwin” (BBCD) Sapienza University of Rome Rome Italy; ^6^ Department of Silviculture Transilvania University of Brasov Brasov Romania; ^7^ LUKE, Natural Resources Institute Finland, Ounasjoentie 6 Rovaniemi Finland; ^8^ Carpathian Widlife Society Zvolen Slovakia; ^9^ Departamento de Biodiversidad y Gestión Ambiental Área de Zoología, Universidad de León León Spain; ^10^ Faculty of Environmental Sciences and Natural Resource Management Norwegian University of Life Sciences Ås Norway

**Keywords:** behavioural plasticity, human infrastructure, large carnivores, movement ecology, telemetry, *Ursus arctos*

## Abstract

In Europe, large carnivore populations have faced a history of persecution and habitat alteration, varying in magnitude across their distribution. Individual animals have developed diverse adaptations to these anthropogenic activities, in most cases to avoid them but in some cases to exploit novel resources in the human‐modified environments they inhabit. Here, we used long‐term GPS‐telemetry data from 108 brown bears *Ursus arctos* collared across three European countries – Finland, Slovakia and Romania—to assess whether the behavioural movement patterns of brown bears are consistent across their range or vary regionally in response to local environmental and anthropogenic influences. We calculated speed, movement direction and daily displacement, and used mixed‐effects models to analyse whether human infrastructure affected brown bear movement behaviour across the study areas. To examine whether the impact of these features varied by study area, and to capture contextual differences that may have affected the movement patterns of bears, we included interactions between environmental predictors and area in the regression models. Our results showed that Finnish bears exhibited consistently higher movement speeds and longer daily displacements than Slovak and Romanian bears, regardless of the proximity to roads, railways, or human settlements. In addition, in proximity to transport infrastructures, Finnish and Slovak bears increased speed, directionality and distance travelled whereas Romanian bears showed the opposite pattern. Conversely, near human settlements, Romanian bears showed higher speeds and less tortuous movements, whereas Finnish and Slovak bears reduced their speed and daily displacements. These contrasting responses suggest that bear movements in multi‐use, human‐modified landscapes are shaped by complex interactions between animal needs and local environmental conditions.

## Graphical abstract

Human activities are major drivers of changes in animal behaviour, resulting in diverse spatial and temporal activity patterns across species. In this study, we analysed telemetry data from brown bears in Finland, Slovakia and Romania, to compare how human infrastructure influences their movement behaviour. The distinct movement patterns observed across the study areas underscores the importance of context‐specific factors in shaping the movement decisions of this large carnivore.

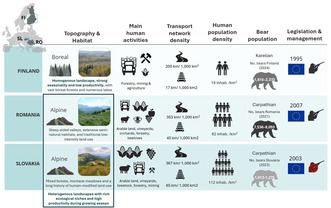

## 1 Introduction

Over time, natural selection has favoured individual animals with behavioural strategies that optimise trade‐offs between resource acquisition and risk avoidance, while maximising fitness benefits (Rosenzweig 1991; Bonnot et al. 2013; Amodio et al. 2019). Additionally, learning and behavioural plasticity have conferred survival advantages to many species in response to environmental challenges (Sol et al. 2005; Parmesan 2006; Amici et al. 2018; Glass et al. 2021). These processes have led to diverse spatial and temporal activity patterns across (Hammond et al. 2018; Rafiq et al. 2023) and within species (Mathot et al. 2011; Delgado et al. 2014; Michelangeli et al. 2019; Kervellec et al. 2023), reflecting the complex interplay between internal drivers and environmental pressures (Pulido 2007; Nathan et al. 2008; Martin et al. 2013).

Different types of human disturbance, such as hunting, leisure activity and infrastructure development, have emerged as major drivers of shifts in behavioural patterns (Foley et al. 2005; Neumann et al. 2013; Januchowski‐Hartley et al. 2014; Newbold et al. 2015). Consequently, many species inhabiting human‐modified landscapes exhibit changes in key ecological processes including foraging strategies (Bowers and Breland 1996; Schloesing et al. 2023), habitat use (Bourbonnais 2018), mating strategies (van de Walle et al. 2019), home ranges (Mertzanis et al. 2005; Elith et al. 2010), and migration patterns (Lamb et al. 2025), reflecting broader behavioural and ecological adaptations to their environments.

Modifications to movement patterns represent one of the most immediate and flexible responses of animals to natural and anthropogenic changes in their environment (Lytle and Poff 2004). Human activities have led to changes in animal movement behaviour and habitat use, across different regions and taxonomic groups (Tucker et al. 2018; Doherty et al. 2021). For example, the presence of physical barriers or other processes leading to habitat fragmentation restricts the ability of animals to move through the landscape, reducing their home range and distance travelled (Tucker et al. 2018; Passoni et al. 2021). The enhancement of resources such as crops, supplemental feeding, and artificial water sources causes further decreases in movements by providing easily accessible food and water (Shannon et al. 2009; Jerina 2012; Jones et al. 2014; Penteriani et al. 2021). However, human presence can also act as a threat, causing animals to increase movements to evade negative encounters (Neumann et al. 2010; Perona et al. 2019; Doherty et al. 2021; Graf et al. 2024).

These short‐ and long‐term behavioural responses can also vary across time, habitats, and among conspecifics (Riotte‐Lambert and Matthiopoulos 2020; Twynham et al. 2021). Large mammals show considerable flexibility in response to environmental changes (Parmesan 2006; Wolff et al. 2020; Fehlmann et al. 2021). In human‐modified landscapes, a common strategy is to increase crepuscular or nocturnal activity to reduce the risk of negative encounters during daylight hours (Gaynor et al. 2018; Zeller et al. 2019; Farhadinia et al. 2020; Mohan et al. 2024; Etana et al. 2025). Mammalian carnivores, in particular, exhibit remarkable behavioural adaptability to balance the opportunities and risks associated with human activities (Bateman and Fleming 2012; Blackwell et al. 2016; Johnson‐Ulrich et al. 2021). For instance, generalist carnivore species living in urban landscapes, such as the coyote *Canis latrans* , northern racoon *Procyon lotor* and red fox *Vulpes vulpes* , have shown a strong spatial affinity towards human features during particular seasons, motivated mainly by increased availability of resources and, in some cases, reduced human persecution in these areas (Prange et al. 2004; Breck et al. 2019; Gelmi‐Candusso et al. 2024; Jackowiak et al. 2024; Burkholder et al. 2025). Moreover, larger predators with more specific diets and need for extensive habitat area showed distinct behavioural patterns in human‐modified landscapes. These ranged from increased occurrence in highly urbanised areas, driven by the search of high energy food sources as observed in big cats (Athreya et al. 2013) and bear species (Dai et al. 2019; Zeller et al. 2019), to pronounced avoidance, as reported for Scandinavian grey wolves born in territories with higher levels of human disturbance (Milleret et al. 2019). These examples illustrate how the life‐history of animals can play a crucial role in shaping individual risk‐taking behaviours, such as the willingness to select areas near human infrastructures or to cross roads (Gibeau et al. 2002; Lewis and Rachlow 2011; Skuban et al. 2017; Milleret et al. 2019; Brogi et al. 2022; Carter et al. 2025).

In Europe, large carnivore populations have a long history of persecution (Zedrosser et al. 2011; Ripple et al. 2014) and continue to be subject to active management in some countries. Therefore, it is reasonable to expect risk‐avoidance strategies towards humans, especially in areas where animals have suffered intense persecution and hunting pressure over centuries (Nellemann et al. 2007; Liberg et al. 2012). Quantitative studies have shown that mortality from hunting, poaching, or retaliatory killings condition the behavioural response of carnivore species to humans (Boitani et al. 2015; Herrero et al. 2021; Hertel et al. 2021; Sanz Perez et al. 2025), with a number of cases focused on the European brown bear *Ursus arctos* (Kaczensky et al. 2006; Ordiz et al. 2011, 2012; Hertel et al. 2016).

The brown bear has problem‐solving capacities (Chambers and O'Hara 2023) serving to adapt and enhance survival and reproductive success in their environment (Rode et al. 2006; Steyaert et al. 2016; Falcinelli et al. 2024; Penteriani et al. 2024, 2025). Avoidance of high‐risk infrastructure is a common response in this species, leading to movement restrictions in areas with higher human footprint and traffic volumes (Skuban et al. 2017; Tucker et al. 2018; Hertel et al. 2025). Conversely, certain contexts can lead bears to use more risky environments, balancing their intrinsic needs with the perceived risk of human presence. For example, female bears with cubs, as well as subadult bears, prioritise safety over foraging opportunities, and utilise areas with human presence as a refuge against both infanticide (Steyaert et al. 2013, 2016) and competitive interactions with dominant conspecifics (Swenson et al. 2001; Elfström et al. 2012, 2014). Bears also take advantage of existing anthropogenic food sources, obtained directly from supplemental feeding used for several purposes, such as hunting or viewing tourism (Kojola & Heikkinen, 2012a; Kavčič et al. 2015; Penteriani et al. 2020, 2021; Cimpoca et al. 2024), or indirectly from unintentionally available resources such as agricultural land and landfills (Skuban 2017; Cimpoca and Voiculescu 2022). In some cases, food conditioning has led individual bears to become habituated to humans, posing a challenge for human‐wildlife coexistence (Elfström et al. 2014; Skuban et al. 2018).

In this study, we utilised a large GPS telemetry dataset of brown bears from three European countries (Finland, Romania and Slovakia), with a small portion of individuals ranging also into northwestern Russia, to examine whether movement patterns varied in response to common human‐related features. Specifically, we analysed brown bear movements in relation to proximity to roads, railways and human infrastructure, as these are the human‐related variables shown to affect the species movements (Kaczensky et al. 2003; Ordiz et al. 2014; Skuban 2017; Skuban et al. 2018; Fedorca et al. 2019; Morales‐González et al. 2020; Thorsen et al. 2022; González‐Bernardo et al. 2023). We also considered topographical and land cover variables, given their recognised importance as ecological determinants of bear movement, independent of human influence (Nellemann et al. 2007; Cimatti et al. 2021; Ashrafzadeh et al. 2023). Additionally, these environmental factors may interact with anthropogenic elements, potentially shaping bear movement patterns (Huber et al. 1998; Štofík et al. 2013; Dickie et al. 2020). We hypothesised that movement patterns would differ among the countries included in our study, as these represent distinct socio‐ecological contexts, with different landscape structure, intensity of human activity, and history of human–carnivore interactions (Falcinelli et al. 2024; Penteriani et al. 2025). Given the significant influence of humans on the behavioural patterns of bears (Kaczensky et al. 2006; Nellemann et al. 2007; Ordiz et al. 2012; Hertel et al. 2016; Morales‐González et al. 2020), we discuss the potential impact of direct and indirect human disturbance in shaping the behavioural response of bears to human infrastructures. Finally, the outcomes of this study will serve to help us better understand bear adaptability in human‐modified landscapes and to outline the importance of context‐specific factors as modulators of the behaviour of this large carnivore. Ultimately, this is essential information to predict future human‐carnivore interactions and draw effective coexistence strategies according to the reality of each socio‐ecological context.

## 2 Materials and Methods

### 2.1 Study Areas and Socio‐Ecological Contexts

**FIGURE 1:**
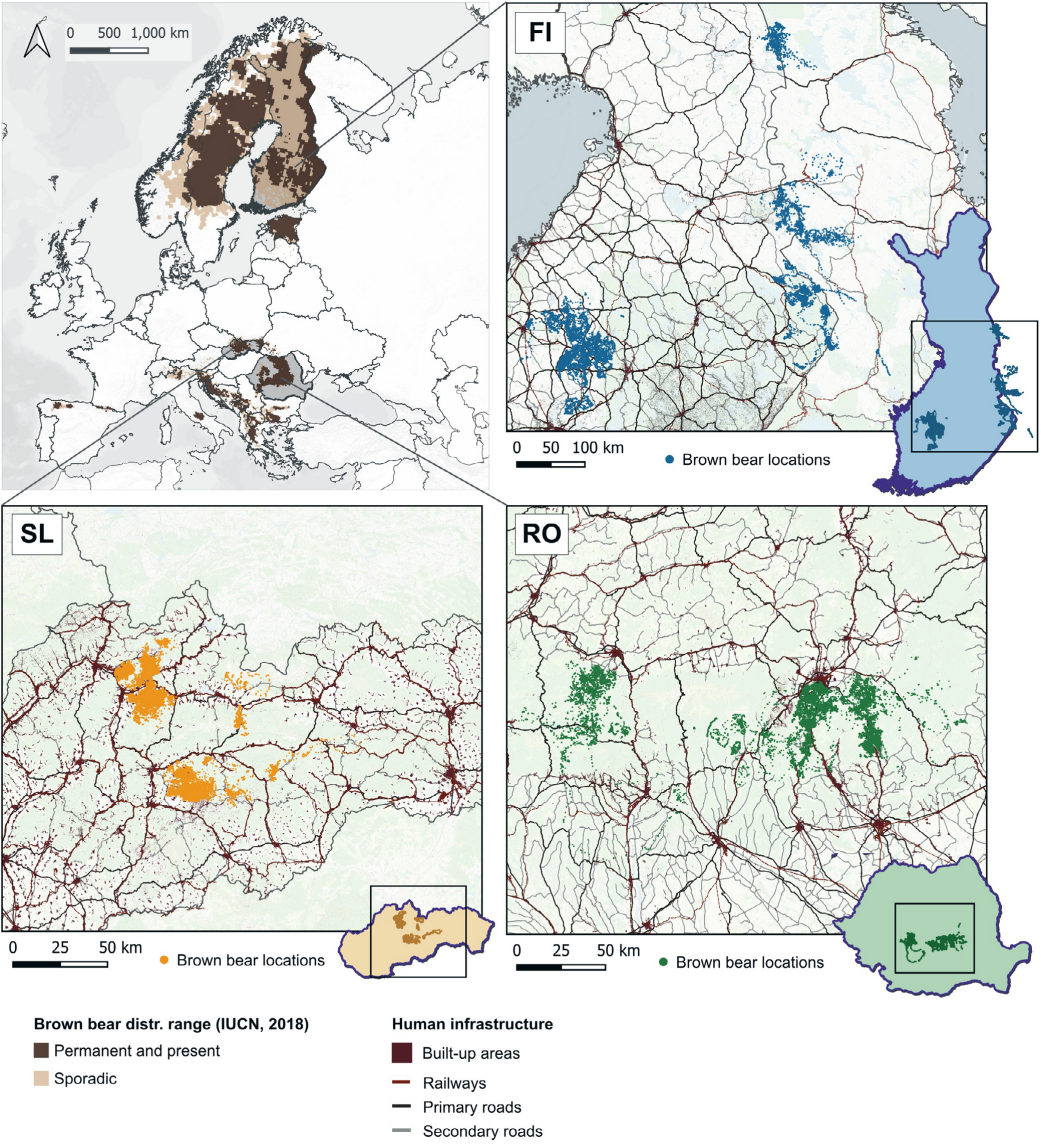
Study areas in Finland (FI), Romania (RO), and Slovakia (SL), including brown bear GPS locations, built‐up areas and major transportation networks within their extents. In the Finnish study area, GPS data is distributed in two main clusters, separated by ~200 km, but these belong to the same population (Kopatz et al. 2012; Keis et al. 2013; Kojola et al. 2020; Olejarz et al. 2022).

This research encompasses three study areas: (1) Southern‐Central Finland and the Finnish and Russian parts of Karelia (hereafter, the *Finnish study area*), (2) the Southern‐Central Carpathian Mountains in Romania (hereafter, the *Romanian study area*), and (3) the Central Slovak Carpathian Mountains (hereafter, the *Slovak study area*) (Figure 1). The Finnish study area is located in the boreal biographical region where the topography is generally flat (elevation range: 80–418 m). The land cover comprises extensive areas of young successional mixed forests resulting from intensive forestry practices, dominated by coniferous and broad‐leaved species (Sundseth et al. 2009), in addition to a mosaic of lakes and wetlands. The study areas in both the Romanian and Slovak Carpathian Mountain range are characterised by a continental and temperate climate. These areas have ample altitudinal ranges compared to Karelia (elevation range: 51–2460 m and 186–2655 m; in the Romanian and Slovak study areas, respectively) with steep to moderate slopes and deep river valleys. These mountainous conditions create various ecosystems, including alpine meadows, heathlands, and natural grasslands at higher elevations, as well as continuous patches of mixed and coniferous forests, transitional woodlands with shrubby vegetation, and rocky areas in lower regions. In the Romanian and Slovak study areas, lowlands and valley bottoms are covered by agricultural lands, including pastures, arable land, orchards, and agroforestry plantations.

**FIGURE 2:**
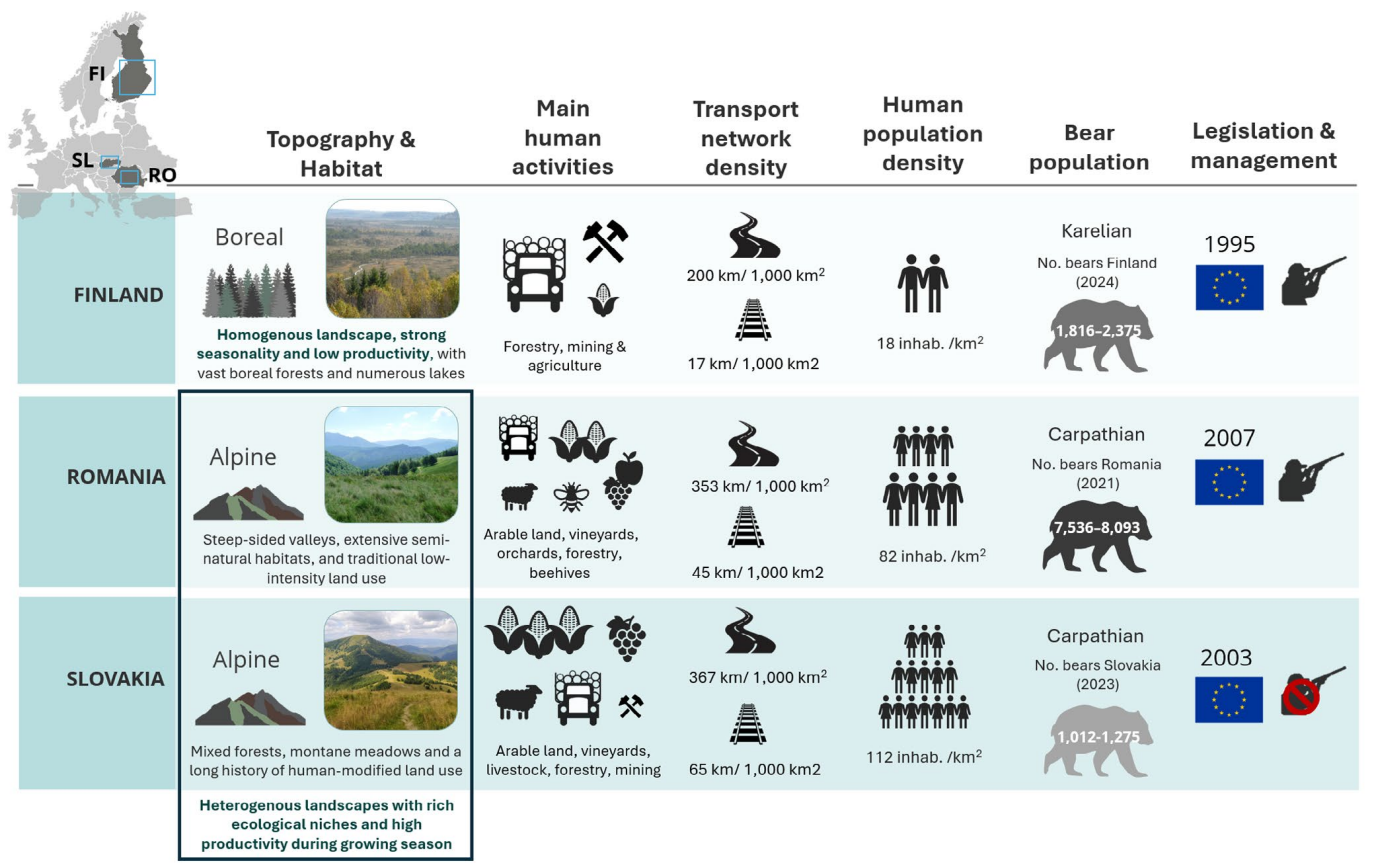
Comparative scheme of environmental and socio‐ecological conditions characterising the primary countries covered by the study areas. Main human activities of the primary sector (icons size proportional to the economic importance) from World Bank Open Data (https://data.worldbank.org/) and Economic Activity (www.economicactivity.org). Transport infrastructures densities from (1) UNECE Transport Statistics Database (https://w3.unece.org/pxweb/en/Table?IndicatorCode=47) and (2) from Hlaváč et al. (2019). Human population densities 2025 from World Bank Open Data (https://data.worldbank.org/). Bear population numbers from reports: Finland: Karhukanta Suomessa 2024 (https://www.luke.fi/fi/uutiset/karhukanta‐on‐kasvussa); Romania: Implementation of the national action plan for the conservation of the brown bear population in Romania 2021 (https://lifeforbear.ro/wp‐content/uploads/2021/11/Action‐plan‐for‐the‐conservation‐of‐brown‐bear‐in‐Romania‐Editura‐Silvica‐2021.pdf); Slovakia: (Tkáčová et al. 2023) (https://www.sopsr.sk/web/?cl=251).

All three study areas are priority regions for brown bear conservation in Europe (Salvatori et al. 2002; Fernández et al. 2012; Kopatz et al. 2012), hosting two viable populations of brown bears that have recovered and expanded their range in recent decades: the Karelian and Carpathian populations (Chapron et al. 2014; Hagen et al. 2015; Swenson et al. 2023). Although the study areas in the Romanian and Slovak Carpathians host a common, genetically distinct brown bear population (Zedrosser et al. 2000; Davison et al. 2011; Matosiuk et al. 2019), the geographical separation and socio‐ecological differences between these areas led us to treat them as two distinct study areas in our analysis (See Figure 2 for outlined differences between the three study areas).

Each of the countries hosting the study areas represent different contexts of human pressure and differing levels of landscape modification that have triggered brown bear behavioural adaptations (Kojola et al. 2006; Rigg & Adamec, 2007a; Penteriani et al. 2022, 2024, 2025; Anuțoiu et al. 2024; Falcinelli et al. 2024). For example, forest logging for commercial purposes and agriculture expansion have been the main drivers of landscape transformation and the cause of brown bear habitat destruction across Finland, Russia, Slovakia and Romania (Swenson et al. 2020, 2023). In Romania, however, despite logging pressures, forests remain comparatively less disturbed, containing one of the largest proportions of old‐growth and primary forests in the EU (Schickhofer and Schwarz 2019; Sabatini et al. 2021).

The expansion of human populations and associated infrastructure is one of the main threats to brown bears in Europe currently (Swenson et al. 2020, 2023). However, the extent and severity of this threat varies across the three study areas due to different human population densities and development trends in the respective countries. In Finland, human population densities decline from Southern‐Central Finland to Karelia (27 and 12 inhabitants/km^2^, respectively; Statistics Finland 2022). These densities are substantially lower than those reported for the Slovak and Romanian Carpathians (79 inhabitants/km^2^ in Slovakia and 70 inhabitants/km^2^ in Romania) (Chabuz 2023), and also lower than the national averages of Slovakia and Romania (See Figure 2). The density of the road network in Finland is 200 km/1000 km^2^ (Schoeters 2023) and the railway density is 17 km/1000 km^2^ (UNECE Transport Statistics Database 2022), whereas in Slovakia and Romania transport networks have higher densities. In Slovakia, the density of roads (367 km/1000 km^2^) and railways (65 km/1000 km^2^) is slightly higher than in Romania (roads: 353 km/1000 km^2^, railways: 45 km/1000 km^2^) (Hlaváč et al. 2019).

In addition to these sources of human disturbance, brown bear populations have faced intense hunting pressure across the three study regions over the past few centuries (Salvatori et al. 2002; Kojola et al. 2006; Štofík et al. 2013). Although the species is strictly protected in Slovakia, bear hunting activity continues in Finland and, starting in 2024, in Romania, where it had been banned since 2016. Harvest quotas in these countries vary by year and management unit according to population demographic rates (Kojola et al. 2003, 2020) and the level of damage caused to livestock and other human‐related activities (Åhman et al. 2022; Ministry of Agriculture and Forestry 2022a).

### 2.2 Bear Captures and Movement Data

Between 2002 and 2024, a total of 108 brown bears (60 females and 48 males) were captured and fitted with GPS‐GSM/Iridium collars in the three study areas to collect movement data (Table 1 and Figure 1). Captures were conducted from spring until bears entered the winter den. Tracking periods for individual bears were limited to a maximum of four consecutive years. The collars had a pre‐programmed drop‐off mechanism with a battery life that lasted, depending on the study area, 1 year or 2 years. When the drop‐off mechanism did not work due to technical issues, the bear was recaptured, and the collar was removed manually. In Finland, permission to capture and manipulate bears was issued by the County Veterinarian of Oulu and the Regional State Administrative Agency of Lahti; captures met the guidelines issued by the Animal Care and Use Committee at the University of Oulu (OYEKT‐6e99), and permits were provided by the provincial government of Oulu (OLH‐01951/Ym‐23). In Romania, permission to capture and manipulate bears was issued by the authorities of the Ministry of Environment, Waters and Forests; captures met the technical guidelines issued by the National Institute for Research and Development in Forestry “Marin Dracea” (INCDS), and permits were provided by the hunting associations (state or private) where the capture took place. In Romania, bears were collared for research purposes. Specifically, they were either brown bears rescued from illegal traps and subsequently released and relocated, or bears captured and relocated due to their presence and problematic activities in urban areas. In Slovakia, permission for capturing and handling the bears was issued by the Ministry of Environment (No. 10155/2010‐2.2). Because there is no ethical clearance of wildlife research in Slovakia, recommendations of the Scandinavian biomedical protocols for capture, chemical immobilisation, and radio‐tagging of brown bears were followed (Arnemo et al. 2017). See Penteriani et al. (2024, 2025) for more details about the data collection process and protocols followed in each study area.

**TABLE 1:** Summary of the brown bear GPS‐telemetry datasets used in this study from Finland, Romania, and Slovakia.

Country	Total period	Annual period of capture	Total no. individuals (♀; ♂)	No. GPS locations collected	No. GPS locations per individual Avg. (±SD)^a^	Fix interval (hours)	Data extent (km^2^)
Finland	2002–2014	April–November	46	35,603	766 (±650)	2	~68,150
			(27; 19)				
Romania	2006–2024	March–December	37	63,004	1687 (±1689)	1–2	~24,000
			(25; 12)				
Slovakia	2008–2018	April–October	25	74,809	2979 (±2180)	1	~6850
			(8; 17)				

^a^ Average and standard deviation in parenthesis.

### 2.3 Landscape Data

We collected spatial information for a set of topographic, landcover and human disturbance variables, which have been reported to influence bear movement behaviour (Kalabér et al. 1992; García et al. 2007; Nellemann et al. 2007; Štofik et al. 2013; Mateo‐Sánchez et al. 2014; Pop et al. 2018; Morales‐González et al. 2020; García‐Sánchez et al. 2021; Bogdanović et al. 2023; Falcinelli et al. 2024). All spatial datasets used to derive these landscape variables were downloaded from open sources (Table A1 and Appendix 1), converted to raster format with a common resolution of 100 × 100 m and re‐projected into the European Coordinate Reference System (EPSG: 3035—ETRS89‐extended/LAEA Europe). Landscape variables derived from the raster layers were extracted at each bear GPS location. The processing and calculation of all rasters were carried out in QGIS version 3.28 (QGIS Development Team 2022), GRASS GIS version 7.8 (GRASS Development Team, 2019), and by the R package ‘*raster*’ (Hijmans et al. 2024).

We calculated the *Terrain Roughness Index* (Riley et al. 1999) from the ASTER Global Digital Elevation Model (NASA/METI/AIST/Japan Spacesystems & U.S./Japan ASTER Science Team 2019) to characterise topographic heterogeneity and to capture its potential influence on animal movement. This variable also influences bear selection of refuge sites, climatic conditions and the distribution of food resources (Nellemann et al. 2007; Ashrafzadeh et al. 2023). We generated a *land cover* map grouping the original 23 classes of the Global Land Cover layer of the Copernicus European programme (GLC 2015) into two unique classes: (1) anthropogenic areas, i.e., all surfaces altered by humans, including urban and cropland areas; and (2) semi‐natural and natural areas, i.e., forests, natural open areas and water body classes. We derived this variable using the Land Cover data from 2015 to avoid adding complexity to our analysis, assuming that this intermediate year of the longest dataset available in the Romanian study areas provided a realistic picture of the landscape conditions for this study. We further calculated the Euclidean distances (in meters) to *main roads*, *secondary roads* and *railways* using the transport infrastructure layers from OpenStreetMaps (OSM 2023); and to *human habitation*, including settlements, villages, and isolated houses, using the Global Human Settlement Layer (GHS‐BUILT‐C 2018). Finally, we considered *human population density* from the GHS population spatial raster dataset of Europe (GHS‐POP 2015; Carneiro et al. 2016) as an indicator of the intensity of human presence and derived activities across the study areas. See Appendix 2; Table A2 for proportions of land cover classes and density of human infrastructures in the study areas, as additional information of the different environmental and anthropogenic contexts of study.

### 2.4 Movement Analyses

#### 2.4.1 Movement Metrics

Bear GPS data was resampled at a consistent fix rate to avoid errors derived from irregular frequencies in the following analyses (Graves et al. 2014). We resampled locations across bear tracks keeping only those locations that were 2 h (±15 min) apart using the ‘*amt*’ R package (Smith et al. 2023). We estimated step lengths (m) and turning angles (rad) between successive locations (Forester et al. 2009) using the ‘*adehabitatLT*’ R package (Calenge 2006). From step lengths, we obtained movement speed (in km/h) and the total and net daily distance travelled (in km) by individual, year and study area. These parameters are commonly used in animal movement research, as they provide valuable information on an individual's movement and response to environmental and anthropogenic factors within the landscape. Speed can explain immediate reactions of bears to their environment, such as avoidance of disturbance factors or active movement behaviours driven by attractors (Valeix et al. 2010; Scrafford et al. 2018; Whittington et al. 2022). Daily total and net distance travelled indicate effects in the longer term, informing us about the navigation capacity of bears and their movement strategies to meet their basic needs (Belotti et al. 2012; Karelus et al. 2017; Johansson et al. 2024). Additionally, turning angles and net distances were used to identify if the animal had a trend of turning back to the same area along a specific time lag (staying on‐site) or if it had a directed movement forward (Gutenkunst et al. 2007; Gelmi‐Candusso et al. 2024).

To evaluate the effect of landscape variables on daily bear movements, we calculated the median value of each landscape variable for each day. We ensured the quality of the data precision by discarding brown bear locations with a dilution of precision (DOP) above 10 (Cargnelutti et al. 2007). Additionally, we examined outliers in detail as those values of movement metrics exceeding biologically realistic thresholds for bears (Weber 1986; Ordiz et al. 2019; Penteriani et al. 2021, 2022; Thorsen et al. 2022), and manually removed them if clearly associated with unrealistic movements (e.g., human intervention of transportation of collars to the installation point or to the relocation point where the bear was released). Although bear species, among other mammals, may exhibit short‐term behavioural changes following collaring events, we retained post‐release GPS locations in our analyses. Given the long temporal span of monitoring per individual and the relatively brief duration of potential post‐capture effects in carnivores (4–7 days; Stiegler et al. 2024), we considered that the influence of capture‐related behavioural changes on our results would be negligible. After filtering the dataset, 172,160 locations remained for analysis.

#### 2.4.2 Statistical Analysis

To analyse the effect of human infrastructures on brown bear movement behaviour across the three study areas, we built four separate models, each using one of the following response variables: speed, total distance, net distance, and turning angle. Model assumptions were checked by visual analyses of the residuals. The response variables speed, total distance and net distance showed a skewed and leptokurtic distribution, but after log‐transforming the data, the residuals were normally distributed. Thus, we fitted the models using a normal distribution (i.e., Linear Mixed effect Models, LMMs). We simplified the response variable ‘turning angle’ into a binary index: 1 for a positive cosine indicating forward movement, and 0 for a negative cosine indicating backward movement (Benhamou 2006). This index (hereafter referred to as ‘direction index’) is commonly used to describe the direction of an individual movement (Turchin 1998; Avgar et al. 2013). As the direction index was a binomial response variable, we built a Generalised Linear Mixed Model (GLMM).

Our predictors were the interactions between the seven landscape variables with the variable ‘study area’. We tested multicollinearity among the predictors by using the variance inflation factors (VIFs; R package ‘car’ v.3.1–3; Fox et al. 2024). We followed a stepwise backward model selection process, removing highly correlated variables (VIF > 10) one at a time and retaining the most relevant variables for the analysis. Due to high multicollinearity, we excluded human population density, landcover type, and distance to main roads. The terrain roughness index introduced high multicollinearity in the models (VIF = 10.42) due to its strong correlation with the study area, reflecting underlying topographic characteristics among the study areas. However, given its significant influence on brown bear movements, particularly in mountainous terrains (Nellemann et al. 2007; Skuban et al. 2018; Cristescu et al. 2019), it remains an essential variable to consider in these analyses. Moreover, the interaction between these variables allows testing if the effect of TRI differs among study areas, rather than assuming a uniform effect. Therefore, the variables that remained in subsequent models were the Terrain Roughness Index and the Euclidian distance to secondary roads, railways and human habitations. All landscape variables selected were standardised using *z*‐score transformation with a mean of 0 and a standard deviation of 1.

**FIGURE A1:**
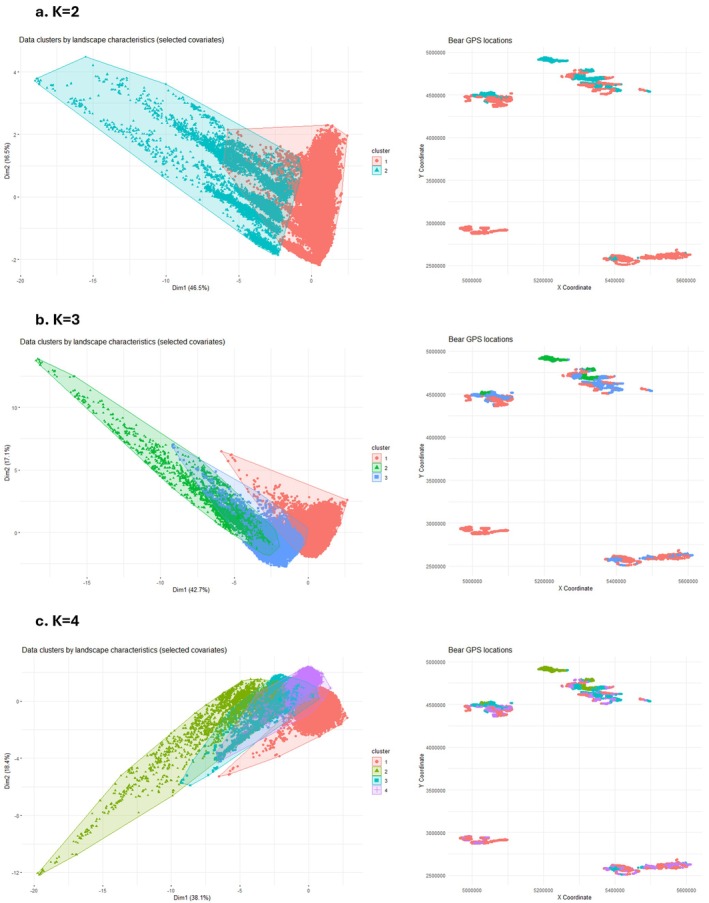
*K*‐means cluster analysis of anthropogenic variables at brown bear locations, with *K* = 2, 3, and 4 clusters. For each *K*, the analysis identifies natural groupings of bear locations based on the anthropogenic variables: Distance to secondary roads, distance to railways, and distance to human habitations. The results depicted in the Figure indicate that data from Karelia and Southern‐Central Finland represent the same natural groupings. This analysis confirms that environmental conditions across the Finnish study area are relatively similar, while showing characteristics not represented in the Carpathian Mountains.

Given the large extent and clustered distribution of the GPS data across the study areas, as is the case especially in Finland (Figure 1), we used a k‐means cluster analysis based on the three selected human‐related predictors to validate that each brown bear population was associated with similar environmental characteristics. This analysis confirmed distinctive anthropogenic scenarios between the Finnish and Carpathian populations, determined by the proximity of infrastructure to areas inhabited by GPS‐collared brown bears. It also revealed notable similarities between the anthropogenic landscape characteristics within each population, despite them being geographically separate (See Figure A1; Appendix 2).

In each model, we included the bear study area, bear ID, and monitoring year as random factors. This approach helped us to deal with unbalanced data and accounts for any potential factors not considered by our predictors that may influence bear movement behaviour related to the study area, individual bears, and the monitoring year. However, the models with the selected predictors showed an over‐parameterisation when both bear ID and study area were included as random effects. To address this issue and reduce model complexity, we retained year as the sole random effect, as recommended by Bolker (2009) and Grueber et al. (2011). This decision was based on the need to balance model complexity with interpretability while adequately accounting for variability in the dataset.

Model fitting and selection were carried out using the R package ‘*MuMIn*’ (Bartón 2024). We built models using all possible combinations of the non‐correlated predictors and ranked them based on the model's Akaike's Information Criterion (AIC; Akaike 1992). We considered all models with ΔAIC < 2 as equally competitive and selected the simplest one as the most parsimonious model. Statistical analyses were performed in R (version 4.2.2; R Development Core Team 2022), and all models were fitted using the R package ‘*lme4*’ (version 1.1–21; Bates et al. 2015). Finally, after fitting the model, we assessed the deviance per degrees of freedom and examined the distribution of residuals using normal Q‐Q plots to ensure unbiased estimates and to validate the selected models.

## 3 Results

**FIGURE 3:**
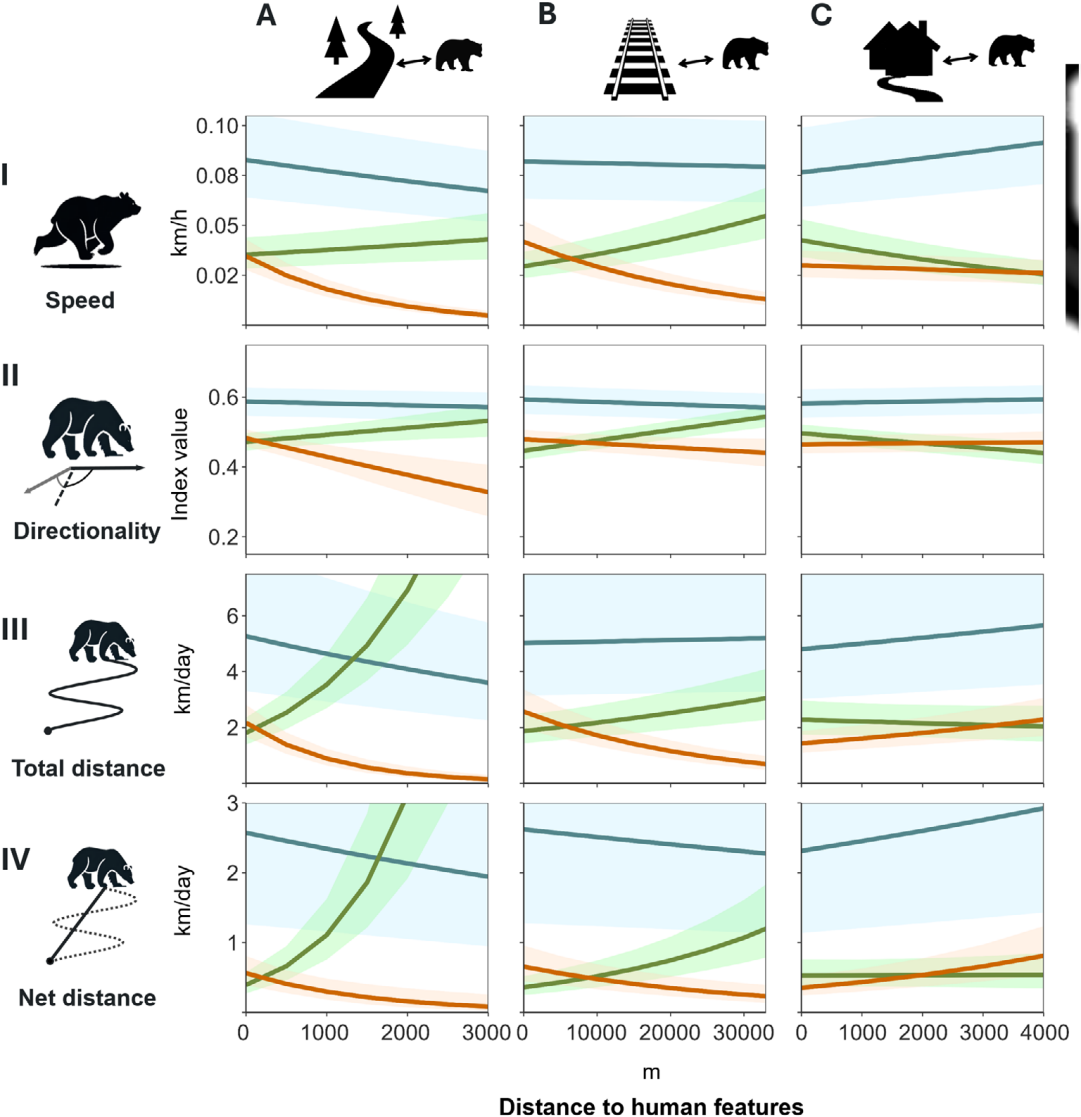
Linear regression lines of the predicted values (back‐transformed) representing the effect of distance to human features (A–C) on brown bear movement variables (I‐IV). Lines are coloured by study area: Blue = Finnish, green = Romanian, orange = Slovak.

Our analyses revealed significant differences in bear movement patterns among the three European study areas (Table 2). In the Finnish study area, bears showed the highest values across the estimated metrics of speed and daily total and net distance travelled (mean, variance, median, maximum value; See Table A3 in Appendix 2), as well as a larger proportion of turning angles, indicating directional movements. These contrasted with Slovak and Romanian bear metrics, where pairwise comparisons revealed no significant differences in speed, turning angle or daily displacement estimates (Table 2; Table A3). The observed variation in the movement metrics among study areas was confirmed by the later model outcomes (Table 3 and Table A4). The speed and the total daily travel distance of Finnish bears were twice as high (Table 2) and more directed than those of the other two study areas, regardless of the effects of the predictor variables (Figure 3).

**TABLE 2:** Summary statistics of the movement parameters of brown bears in three European study areas, including mean values and standard deviations of speed, turning angle and daily total and net distance travelled; and the percentages of direction index values. See also Table A3 for additional statistics of movement and landscape variables.

Study area	No. bears	Monitoring days	Speed (km/h)	Turning angle (rad)	Direction index (0%; 1%)	Total distance travelled (km/day)	Net distance travelled (km/day)
FI	46	3305	0.33 ± 0.51	−0.003 ± 1.764	45; 55	7.56 ± 6.24	4.01 ± 4.67
RO	37	5666	0.17 ± 0.31	−0.011 ± 1.903	53; 47	3.96 ± 3.62	1.80 ± 2.48
SL	25	7496	0.16 ± 0.27	−0.006 ± 1.886	52; 48	3.71 ± 3.05	1.52 ± 1.76

Abbreviations: FI, Finnish study area; RO, Romanian study area; SL. Slovak study area.

**TABLE 3:** Beta estimates, 95% confidence intervals and statistical significance for the predictor variables included in the top mixed models to evaluate movement patterns of brown bears in three European study areas: Distance to secondary roads (DSR), distance to railways (DRW), distance to human habitations (DHH), Terrain Roughness Index (TRI). The Finnish study area (FI) was used as the baseline (intercept) to compare relationships between predictors and movements in the Romanian (RO) and Slovak (SL) study areas. Each column presents the results of each top model assessing the effect of conditions specific to each study area in 2‐h and daily movement parameters. We used predictor values for 2‐h observations for fitting model speed and direction index; and medians of predictor values by day for fitting model total and net distance travelled. All predictor variables were standardised using *z*‐score transformations to fit the models.

Predictors	Speed	Direction index	Daily total distance	Daily net distance
	exp (*β*)	*β*	exp (*β*)	exp (*β*)
Intercept	0.08 (0.06–0.10)***	0.35 (0.18–0.51)***	5.08 (3.20–8.07)***	2.51 (1.23–5.09)*
RO	0.44 (0.39–0.51)***	−0.44 (−0.58 to −0.30)***	0.43 (0.29–0.64)***	0.21 (0.11–0.39)***
SL	0.35 (0.31–0.40)***	−0.48 (−0.62 to −0.34)***	0.33 (0.22–0.49)***	0.19 (0.10–0.35)***
DSR	0.97 (0.96–0.98)***	−0.01 (−0.02–0.00)	0.94 (0.92–0.97)***	0.96 (0.92–1.00)*
DRW	0.99 (0.97–1.01)	−0.03 (−0.05 to −0.02)***	1.01 (0.98–1.05)	0.95 (0.90–1.00)
DHH	1.07 (1.06–1.09)***	0.02 (0.00–0.04)*	1.07 (1.04–1.10)***	1.1 (1.05–1.15)***
TRI	1.29 (1.16–1.44)***	0.14 (0.03–0.25)*	1.19 (0.89–1.59)	1.76 (1.11–2.79)*
RO:DSR	1.07 (1.04–1.10)***	0.05 (0.02–0.08)**	1.46 (1.37–1.56)***	1.7 (1.54–1.89)***
SL:DSR	0.76 (0.72–0.80)***	−0.09 (−0.15 to −0.04)**	0.7 (0.62–0.78)***	0.77 (0.65–0.93)**
RO:DRW	1.25 (1.21–1.30)***	0.17 (0.13 to 0.21)***	1.17 (1.09–1.26)***	1.6 (1.43–1.80)***
SL:DRW	0.67 (0.63–0.71)***	−0.02 (−0.08 to 0.04)	0.62 (0.56–0.70)***	0.73 (0.61–0.88)**
RO:DHH	0.76 (0.73–0.79)***	−0.11 (−0.16 to −0.06)***	0.9 (0.81–0.98)*	0.92 (0.79–1.07)
SL:DHH	0.88 (0.85–0.92)***	−0.01 (−0.05 to 0.03)	1.13 (1.04–1.22)**	1.27 (1.12–1.45)***
RO:TRI	0.53 (0.48–0.59)***	−0.28 (−0.39 to −0.17)***	0.59 (0.44–0.80)***	0.42 (0.26–0.67)***
SL:TRI	0.49 (0.44–0.55)***	−0.25 (−0.37 to −0.14)***	0.53 (0.40–0.71)***	0.35 (0.22–0.56)***
Marginal *R* ^2^/Conditional *R* ^2^	0.047/0.106	0.008/0.022	0.120/0.297	0.069/0.217

*Note:* Significance levels: () *p* ≥ 0.05; (*) *p* < 0.05; (**) *p* < 0.01; (***) *p* < 0.001.

The most parsimonious models included a significant interaction between the environmental predictors and study area, indicating that their effects on movement variables varied by study area (See Table A5 in Appendix 3 for the five top competing models and their parameters). Moreover, these model outputs supported expected differences in brown bear movement patterns in response to human infrastructures (Figure 3 and Table 3). Finnish and Slovak bears gradually increased speed, total and net distance travelled near secondary roads, whereas Romanian bears exhibited a slight decrease in speed and a more pronounced reduction of total and net daily displacement as they moved closer to infrastructures (Figure 3A). Opposite effects were also observed between Slovak and Romanian bears in relation to the presence of railways in all movement parameters: Romanian bears showed minimum speed, directionality and daily total and net distance travelled, whereas Slovak reached maximum values. Distance to railway had less impact on the movement patterns of the Finnish bear population compared to those in the Carpathian regions (Figure 3B). Additionally, in Romania, bears were more likely to change direction when located closer to secondary roads and railways compared to the other study areas (Figure 3, panels AII‐BII).

In contrast to the effects observed in response to linear infrastructure, Finnish bears reduced their speed and their total and net travel distance when they were closer to human habitation. Despite minor variations within the Carpathian region, bears in Slovakia and Romania generally maintained consistent movement patterns across the full range of distance to human habitation (Figure 3C). The influence of these human features appeared to be more pronounced in Romanian bear speed and directionality, with a negligible effect in their total and net daily distance travelled compared to Slovak bears (Figure 3, CI). Romanian bears showed higher directionality near human habitation, meaning less tortuosity and variation in their movement path (Figure 3, CII). In comparison, Finnish and Slovak bears did not show significant changes in movement direction in response to this human infrastructure (Figure 3, CII). On the other hand, Slovak bears gradually decreased daily total and net distance travelled near human habitation, as also was observed in Finland (Figure 3, CIII‐CIV).

**FIGURE A2:**
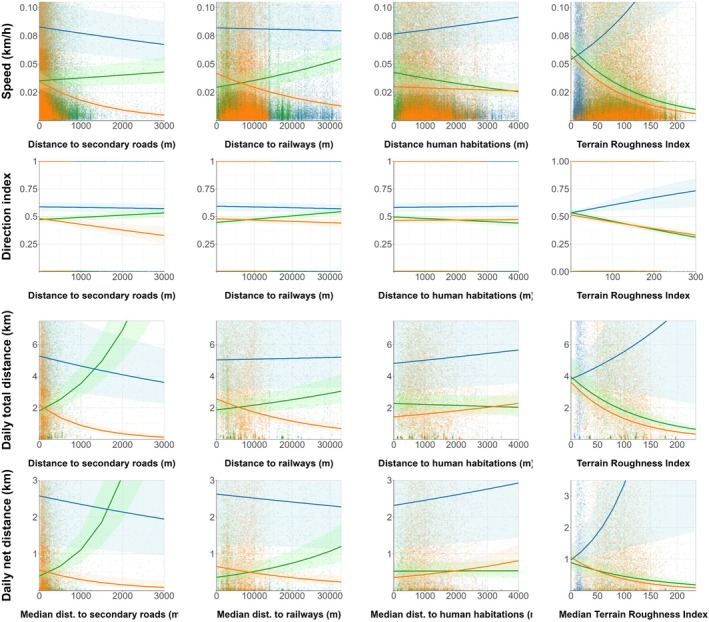
Linear regression lines of the predicted values (back‐transformed) and original data points for brown bears in our study. Lines are coloured by study areas: Blue = Finnish, green = Romanian, orange = Slovak.

Regarding other landscape factors, model results indicated a significant impact of terrain roughness on bear movement across the three study areas. As the terrain roughness index increased, bears in the two Carpathian study areas exhibited slower, shorter, and more tortuous movements, in contrast to the faster and longer total and net daily movements observed in the most rugged terrains of the Finnish study area (See Figure A2, Appendix 3). Moreover, Finnish bear locations generally occurred at greater distances from secondary roads, with a maximum value of 11,379 m, whereas bears in the Carpathian study areas were never found further than 975 m and 2990 m from these types of roads (see data overdispersion at maximum values of the predictor variables in Figure A2). Finally, the residual deviance across all models indicated unexplained variability in the data, suggesting that additional factors may have influenced bear movement patterns.

## 4 Discussion

Using movement data from three study areas across Europe, we identified varying behavioural patterns within the European range of this species, supporting our initial hypotheses. First, we observed significant differences in movement speed and distances travelled between the Finnish and Carpathian study areas. Second, brown bears seemed to display contrasting behavioural responses in relation to human infrastructures across the three study areas. These findings suggest that varying environmental conditions and levels of disturbance shape brown bear movement decisions, leading to local behavioural adaptations to both human features and environmental variables.

Finnish bears generally travelled further distances at higher speeds compared to Slovak and Romanian bears, which showed similar movement metrics. The flatter terrains and lower densities of humans and their infrastructures in Finland posits less movement constraints compared to the Carpathians (Barry et al. 2020; Thorsen et al. 2022). In contrast, bears in the Carpathians live in more complex landscapes, characterised by varied and rougher topography and higher human infrastructure densities (Van Maanen et al. 2006; Cimatti et al. 2021). The heterogeneity of these mountainous landscapes generally implies higher energy costs to move longer distances, consistent with topography effects observed in Slovak and Romanian bear movements in our models. Comparable patterns have been reported in previous studies, showing that areas with constant and predictable resource availability, such as the Carpathians, allow shorter and slower movements and home range sizes (Rigg & Adamec, 2007a; Pop et al. 2018; Riotte‐Lambert and Matthiopoulos 2020; Hertel et al. 2025). Contrastingly, in areas with stronger seasonality and lower productivity, such as the boreal ecosystems, brown bears and other large mammals have larger home ranges (Nagy and Haroldson 1990; Swenson et al. 1998; Hertel et al. 2025) and often exhibit increased movements to obtain sufficient food (Mcloughlin et al. 2000; Dahle and Swenson 2003; Morellet et al. 2013; Tucker et al. 2018; Hertel et al. 2019).

Differences in the average female home range sizes reported in the Finnish (581 km^2^; Olejarz et al. 2022), Slovak (136 km^2^; Zięba and Zwijacz‐Kozica 2005; Rigg & Adamec, 2007a) and Romanian (120 km^2^; Pop et al. 2018) study areas support the influence of seasonal abundance and richness of food resources on bear movement and space use. Moreover, these patterns may also be related to brown bear density and its variation among study areas. Lower densities in Finland (0.3–1.8 bears/100 km^2^, values converted from original units of bears/1000 km^2^; Kojola et al. 2003; Schregel et al. 2012) compared to Slovakia (4–10 bears/100 km^2^; Rigg & Adamec, 2007a; Tkáčová et al. 2023) and Romania (7.5–14.8 bears/100 km^2^; Popescu et al. 2017) could explain the ability of Finnish bears to travel longer distances than bears living in the Carpatian population. In the Carpathian study areas, higher bear densities might restrict the availability of space for solitary habitat use (Zięba and Zwijacz‐Kozica 2005), thereby limiting the net distances travelled by individuals (Schulte et al. 2021). This interpretation aligns with findings from Finland, where smaller home range sizes were recorded in areas with higher brown bear densities, attributed to reduced land availability for bear individuals and increased exposure to risks such as encounters with infanticidal males and hunters (Olejarz et al. 2022). However, our analysis did not explore the relationship between these variables and movement behaviour, and our interpretation of their influence should be considered tentative.

Although our results confirmed population‐level adaptations to environmental conditions, our models also revealed contrasting behavioural responses to human infrastructures. Interestingly, despite the contrasting topography and infrastructure density in Finland and Slovakia, bears in both study areas exhibited similar behavioural responses to secondary roads, railways, and built‐up areas. In contrast, bears in the geographically closer study areas of Slovakia and Romania displayed markedly different patterns, with opposite effects of infrastructure on their speed, directionality, and total and net daily distance travelled. These findings suggest that, in addition to terrain ruggedness and infrastructure presence, other context‐specific factors play a significant role in shaping bear behaviours near human features. These additional factors may be related to the different socioecological pressures and opportunities for bears associated with human infrastructure in each study area.

Previous research has interpreted faster and more directed movements near infrastructure either as a response to perceived higher levels of risk (Skuban et al. 2017; Carter et al. 2025) or as a strategy to exploit linear features as efficient movement corridors (Mattson 1990; Gibeau et al. 2002; Roever et al. 2010; Dickie et al. 2020). In our study, bears in the Finnish and Slovak study areas increased their movement speed, directionality and travel distance near secondary roads and railways, yet the similar patterns observed may have arisen from different context‐specific drivers. In Slovakia, for example, bears are known to be attracted to anthropogenic food sources located near human infrastructure, such as agricultural crops and orchards, resulting in shifts of their spatial and temporal activity patterns (Skuban et al. 2018; Find'o et al. 2019). These bears often move from forest with poor herbaceous cover to agricultural land, increasing road‐crossing frequency during periods of low human activity (Find'o et al. 2007; Skuban et al. 2016; Skuban 2017). These patterns have been also observed in human‐modified landscapes offering concentrated and accessible resources by other wildlife species with plastic behaviours (Beckmann and Berger 2003; Mumme et al. 2023). In contrast, Finnish bears inhabit less fragmented landscapes with lower human pressures, given the low human population densities and minimal urban expansion typical of many rural and wilderness areas across the Finnish study area (See Figure 2 and Table A2). Yet, their increased movement near infrastructure suggests they may perceive these areas as risky but necessary to traverse in order to access higher‐quality foraging patches (Hertel et al. 2019; Penteriani et al. 2022). In both study areas, maximum movement metrics likely reflected a trade‐off strategy, where bears needed to cross human‐modified areas to maximise foraging efficiency in resource‐poor or fragmented habitats (Kuemmerle et al. 2007; Graham et al. 2010; Dickie et al. 2020), while mitigating the mortality risks associated with transport infrastructure (Proctor et al. 2019; Torretta et al. 2023).

In contrast to movement patterns observed in the Finnish and Slovak study areas, our results showed reductions in Romanian bear movements and higher chances of changing direction close to transport infrastructure. Although the national densities of transport infrastructures are similar in Slovakia and Romania, only a fraction of the Romanian network lies in the mountain range. In fact, compared to high traffic volumes on highways and other roads in the Slovak Carpathians (maximum AADT in E‐Roads: 28,000 vehicles/day; UNECE 2005; Skuban et al. 2017; Find'o et al. 2019), no major highways cross the brown bear range in the Romanian Carpathians. Consequently, the national and county roads in the Romanian study area experience high traffic intensity (maximum AADT in E‐Roads: 22,000 vehicles/day; UNECE 2005; Fedorca et al. 2019; Domokos et al. 2024). Moreover, in the Romanian Carpathians, roads and railways generally run through valleys, leaving steep, inaccessible, forest‐covered slopes on either side. The parallel placement of railways and main roads with high traffic volumes linking touristic areas creates combined linear barriers that limit individual bear movements, as has been shown by reduced gene flow (Fedorca et al. 2019) and high numbers of bear‐vehicle collisions (Fedorca et al. 2021) in the Romanian Carpathians. Our findings are consistent with these inferences, suggesting that transport infrastructures may act as restrictive elements for bears in Romania, likely due to high perceived risk.

Alternatively, we speculate that different bear densities, sex ratios and levels of human intervention between the Carpathian study areas might explain the contrasting movement patterns we found. Compared to the Slovak scenario, higher bear densities in Romania (Van Maanen et al. 2006; Popescu et al. 2017; Stăncioiu et al. 2019) may lead vulnerable bears, i.e., subdominant individuals or females with cubs, to inhabit areas closer to traffic infrastructures (Morales‐González et al. 2020). Once in these areas, subadult males and females with cubs may further select locations near roads to reduce the risk of negative encounters with dominant conspecifics, as observed in other European populations (Kaczensky et al. 2006; Nellemann et al. 2007; Elfström and Swenson 2009; González‐Bernardo et al. 2023). This pattern aligns with the human shield hypothesis, which suggests that bears use areas with higher human activity as a protective buffer (Steyaert et al. 2016; Dickie et al. 2020; Gaynor et al. 2025). Furthermore, this is supported by the higher likelihood of female bears' occurrence near human settlements in Romania (García‐Sánchez et al. 2021; Domokos et al. 2024).

Unlike the strong effect observed in response to distance to roads for all study areas, we found a weaker relationship between distance to human habitation in total and net daily displacements, especially in the Romanian bears. The heterogeneity of human settlements in the Romanian Carpathians, from more developed urban areas to remote villages, could lead to varying movement patterns at the same distance from human habitations. Furthermore, other variables or mechanisms associated with these built‐up areas might be driving diverse behavioural responses (e.g., presence of artificial sources of food, suitable habitat areas, human attitudes towards bears). Faster movements are likely driven by both attraction to artificial sources of food close to human settlements, where scavenging behaviours are common in some towns within the Romanian study area (Cotovelea et al. 2015; Cimpoca and Voiculescu 2022; Cimpoca et al. 2024), and by the perceived increased risk derived from deterring techniques and guardian dogs in these areas (Pop et al. 2012). In such contexts, bears that feed on garbage are often disturbed, forcing them to move rapidly through towns and their surroundings in search of food while remaining alert to potential threats. However, the short‐term responses reflected in Romanian bear speeds are not translated into daily total and net distance travelled. Based on these insights, we inferred the predictability of risk associated with human settlements was lower than that with proximity to roads in Romania.

In the Finnish and Slovak study areas, we observed a significant reduction in daily mobility close to built‐up areas, indicating consistent traits in bear responses between the regions. Skuban et al. (2018) found that Slovak bears are drawn to settlements, finding resource‐rich and quiet refuges in agricultural lands with shrubby and young forest. Alternatively, higher levels of human activity in the surrounding urban environments could limit the willingness of bears to enter human habitations. In comparison with Slovakia, bears in Finland are more easily able to retreat from people due to lower human population densities and associated features, avoiding longer displacements in urbanised areas (see data distribution across distance to human habitations in Figure A2). Further analysis should integrate prediction of the selection preferences in relation to the considered infrastructures, to gain valuable insights for the interpretation of our results and a further understanding of human‐bear coexistence and connectivity. Likewise, further studies should look at the potential effect of sex and age classes on the speed, distance travelled and directionality of movements by bears, to better understand their behaviour in relation to human infrastructures in the different populations.

Among other context‐dependent factors, the association of anthropogenic stimuli with either negative or positive experiences across the study areas may shape individuals to display wary or bold behaviours towards human infrastructures (Frid and Dill 2002; Darrow and Shivik 2009; Francis and Barber 2013). Moreover, previous research has shown that the mere fear of humans explains key changes in carnivore behaviours in response to human activities and presence (Ordiz et al. 2011, 2013; Suraci et al. 2019). Long‐term persecution and present anthropogenic disturbances, such as poaching and hunting, can heighten fear responses and elevate risk perception in bears (McLellan and Shackleton 1989; Rode et al. 2006; Ordiz et al. 2011, 2012). Supporting the concept that past experiences influence future responses, previous research on carnivore species has found that lower harvest rates are associated with higher levels of road mortality (Tri et al. 2017; Logan and Runge 2021; Moore et al. 2023).

Our findings suggest that in Europe, where brown bears have experienced varied histories of persecution and management over centuries, behavioural responses to human presence may be shaped not only by current disturbances but also by long‐term socio‐ecological contexts. Different levels of pressure experienced in the past across the species range may have led to avoidance behaviours in certain regions, whereas in others, prolonged exposure to human presence and predictable anthropogenic food sources may have led to increased habituation. Historical records indicate that intense persecution of brown bears during the first half of the 20th century caused severe declines across the European countries in our study (Koreň et al. 2011; Chapron et al. 2014; Mykrä & Pohja‐Mykrä, 2015a). In Romania, early restrictive measures implemented in the early 1950s led to a substantial increase in bear numbers, peaking at almost 8000 individuals by 1988 (Jurj et al. 2021), whereas bears in Slovakia (Find'o et al. 2007) and Finland (Kojola et al. 2003, 2006; Kaczensky et al. 2013a) recovered more gradually. Over the past few decades, a shift towards conservation‐oriented management in Europe has led to the recovery of brown bear populations (Zedrosser et al. 2011), although the timing and implementation of these measures varied across European countries. In Finland and Romania, hunting continues under strict regulations, with quotas adjusted by authorities, recently raised to prevent human‐bear conflicts. However, although the bears are strictly protected in Slovakia, both Carpathian countries are facing challenges with continuous bear population growth, lack of effective management practices, increased poaching, and the implementation of controversial conflict prevention measures like “protective shooting” protocols (See Appendix 4 for additional information on the evolution of brown bear numbers and hunting regulations in the study areas).

The differing historical trajectories and management practices across the countries may have shaped a variety of human behaviours in regions inhabited by brown bears. These human responses, ranging from tolerance to enmity, can exert a significant influence on bear movement patterns, particularly in how individual bears balance risk‐taking and avoidance behaviours in relation to human infrastructures. Consequently, the behavioural adaptations of bears to specific social and environmental contexts are likely reflected in the variation in movement responses to human infrastructure observed across our study areas. The movement patterns identified in this study highlight an urgent need to improve our knowledge on how local contexts may affect behavioural decisions and, consequently, address management measures based on local circumstances.

Research capturing variances in behavioural responses to human disturbances across species ranges remains limited, largely due to data constraints. Our multi‐country dataset allowed us to investigate different adaptation strategies of brown bears to diverse socio‐ecological contexts. Additionally, our findings confirmed the brown bear is an excellent species to explore diverse behaviours in changing environments (Benson‐Amram et al. 2016; Smaers et al. 2021). The attraction of bears to human areas, possibly driven by resource availability, raises concerns about increased mortality risks, road kills, and the emergence of ecological traps (Lamb et al. 2017; Penteriani et al. 2018). In the Carpathian Mountains, artificial food sources attracting bears to urban areas can cause concerns for potential human‐bear conflicts in local communities and trigger management interventions with uncertain long‐term conservation outcomes. Furthermore, regional developments and urban expansion pose significant challenges to brown bear connectivity, potentially disrupting dispersal and gene flow. Finally, the identification of context‐dependent behaviours reinforces the urgency of integrating movement ecology into landscape planning to mitigate the impacts of future infrastructure developments on wildlife. This is particularly important for large carnivores, whose typically large home ranges are vulnerable to habitat fragmentation and potential barrier effects.

## Author Contributions

**Pino García‐Sánchez:** conceptualization (equal), data curation (equal), formal analysis (lead), investigation (lead), methodology (equal), writing – original draft (lead), writing – review and editing (lead). **Vincenzo Penteriani:** conceptualization (equal), funding acquisition (equal), investigation (supporting), methodology (equal), project administration (equal), supervision (supporting), writing – original draft (supporting), writing – review and editing (supporting). **María del Mar Delgado:** conceptualization (equal), data curation (equal), formal analysis (supporting), investigation (supporting), methodology (equal), supervision (supporting), writing – original draft (supporting), writing – review and editing (supporting). **Daniele Falcinelli:** data curation (equal), investigation (supporting), writing – review and editing (supporting). **Ancuta Fedorca:** conceptualization (equal), funding acquisition (equal), investigation (supporting), resources (equal), supervision (supporting), writing – original draft (supporting), writing – review and editing (supporting). **Louise K. Gentle:** conceptualization (equal), investigation (supporting), supervision (supporting), writing – original draft (supporting), writing – review and editing (supporting). **Ilpo Kojola:** funding acquisition (equal), investigation (supporting), resources (equal), writing – review and editing (supporting). **Samuli Heikkinen:** funding acquisition (equal), investigation (supporting), resources (equal), writing – review and editing (supporting). **Slavomír Find'o:** funding acquisition (equal), investigation (supporting), resources (equal), writing – review and editing (supporting). **Michaela Skuban:** investigation (supporting), resources (equal), writing – review and editing (supporting). **Mihai Fedorca:** investigation (supporting), resources (equal), writing – review and editing (supporting). **Ovidiu Ionescu:** investigation (supporting), resources (equal), writing – review and editing (supporting). **Georgeta Ionescu:** investigation (supporting), resources (equal), writing – review and editing (supporting). **Ramon Jurj:** investigation (supporting), resources (equal), writing – review and editing (supporting). **Marius Popa:** investigation (supporting), resources (equal), writing – review and editing (supporting). **Andrés Ordiz:** funding acquisition (equal), investigation (supporting), writing – review and editing (supporting). **Jon E. Swenson:** funding acquisition (equal), investigation (supporting), writing – review and editing (supporting). **Antonio Uzal:** conceptualization (equal), investigation (supporting), methodology (equal), project administration (equal), supervision (lead), writing – original draft (supporting), writing – review and editing (supporting).

## Funding

This work was supported by Nottingham Trent University. European Regional Development Fund. Dr. Joachim and Hanna Schmidt Stiftung für Umwelt und Verkehr, Germany. MCIN/AEI/10.13039/501100011033 and European Union. Romanian National Authority for Scientific Research and Innovation, Nucleu Programme PN 23090304. IDE/2024/000779; SEKUENS and EU funds. Finnish Ministry of Agriculture and Forestry.

## Ethics Statement

This study was performed in line with the principles and guidelines of each country where the data was collected: Finland. Permission to capture and manipulate bears was issued by the County Veterinarian of Oulu and the Regional State Administrative Agency of Lahti (Finland). The capturing of bears met the guidelines issued by the Animal Care and Use Committee at the University of Oulu (OYEKT‐6e99), and permits were provided by the provincial government of Oulu (OLH‐01951/Ym‐23). Slovakia. The Ministry of Environment of the Slovak Republic issued the permit (No. 10155/2010–2.2) for capturing and handling the bears. Because ethical clearance for wildlife research is not required in Slovakia, we adhered to the recommendations outlined in the Scandinavian biomedical protocols for capturing, chemically immobilising and radiotagging brown bears (Arnemo et al. 2017). Romania. Permission to capture and manipulate bears was issued by the Ministry of Environment, Waters and Forests of Romania (No. 1662/23.08.2016). These bears were collared for research purposes, specifically either as individuals rescued from illegal traps and subsequently released and relocated, or as bears captured and relocated due to their presence and problematic activities in urban areas. The capturing of bears met the guidelines issued by the National Institute for Research and Development in Forestry Marin Dracea, INCDS (see technical reports of the project LIFE FOR BEARdProject LIFE 13 NAT/RO/001154; http://www.forbear.icaswildlife.ro/en/), and permits were provided by the hunting associations (state or private) where the capture took place. The overall project received favourable opinion from Nottingham Trent University, School of Animal, Rural and Environmental Sciences, Research Ethics Committee (Project ID 1856072).

## Conflicts of Interest

The authors declare no conflicts of interest.

## Figures and Tables

**FIGURE A3:**
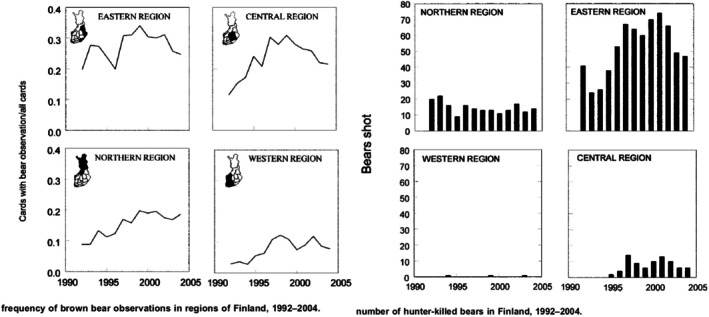
Frequency of brown bear observations (left) and number of bears harvested (right) in different regions of Finland from 1992 to 2004. Figures from Kojola et al. (2006).

**FIGURE A4:**
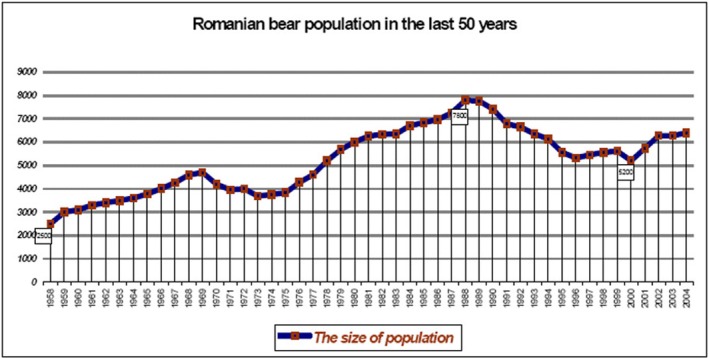
Development of the brown bear population size in Romania (1958–2004). Figure not publised from O. Ionescu.

**FIGURE A5:**
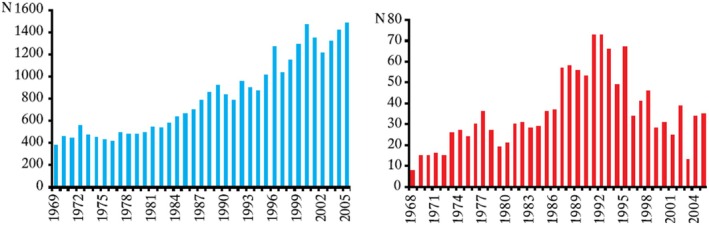
Bear numbers (blue bars) and bears harvested by hunters (red bars) in Slovakia from 1968 to 2005. Graphs from Find'o et al. (2007).

## Data Availability

The data that support the findings of this study are openly available in Figshare at: https://doi.org/10.6084/m9.figshare.30128932.v2.

## Appendix 1 Description and Resources of the Landscape Data Used in the Study

**TABLE A1:** Environmental information used in the study, classified in three categories: (1) topographic, (2) land cover and (3) human disturbances. Using this information, we derived the landscape variables integrated in the analysis.

Class	Spatial data	Description	Type	Resource	Orig. resolution (m)	Year
Topography	Digital Elevation Model	—	*Continuous*	ASTER Global Digital Elevation Model 1 arc second [ASTGTMv003] – NASA	30	2013
Landcover	Land cover	Reclassified in 5 classes: (1) Anthropogenic, (2) forest, (3) open areas, (4) shrubs and water	*Categorical*	Global Land Cover layer (GLC) of the Copernicus programme	100	2015
Human disturbances	Main roads	Highways and other important traffic routes (mostly paved)	*Continuous*	Open Street Map (OSM) road data/Geofabrik roads	Line vector	2023
	Secondary roads	Minor traffic routes (paved and unpaved), including streets, access roads, forest roads, and paths	*Continuous*			
	Railways	Light rail and mainline railways	*Continuous*			
	Built‐up surfaces	Number of square metres occupied by settlements, villages or isolated houses	*Continuous*	Global Human Settlement Layer (GHS‐BUILT‐S)	10	2018
	Human population densities	Number of inhabitants	*Continuous*	Global Human Settlement Layer (GHS POP)	100	2015

## Appendix 2 Landscape and Brown Bear Data Comparison Across the Study Areas: Summary Statistics and Cluster Analysis

**TABLE A2:** Land cover composition, road infrastructure density, and built‐up area density within the Finnish, Romanian, and Slovak study areas. Land cover proportions are expressed as percentages of the total study area, calculated as the number of raster cells per land cover class relative to the total number of cells. Road densities represent the total length of each road type (km) per square kilometre of study area (km/km^2^). Built‐up area densities represent the proportion of built‐up raster cells (GHS values > 0) per square kilometre of study area (km^2^/km^2^).

Study area	Land cover proportion (%)		Road density (km/km^2^)			Built‐up density (km^2^/km^2^)		
	Anthropogenic areas	Semi‐natural and natural areas	Main roads	Secondary roads	Railways	Open spaces with vegetation	Residential buildings	Commercial/industrial uses
Finnish	1.4	98.6	0.07	2.26	0.04	0.0019	0.0001	0.0001
Romanian	18.6	81.4	0.09	1.04	0.07	0.0122	0.0022	0.0004
Slovak	14.4	85.6	0.1	1.73	0.26	0.0093	0.0017	0.0006

**TABLE A3:** Summary statistics of brown bear movement parameters and landscape variables for the three European study areas in Finland, Romania and Slovakia.

	Mean ± SD			Median			Range		
	Finland	Romania	Slovakia	Finland	Romania	Slovakia	Finland	Romania	Slovakia
Movement variables									
Speed (km/h)	0.33 ± 0.51	0.17 ± 0.31	0.16 ± 0.27	0.10	0.03	0.03	0.00–5.57	0.00–4.30	0.00–2.65
Turning angle (rad)	−0.003 ± 1.764	−0.011 ± 1.904	−0.006 ± 1.887	0.000	−0.016	−0.010	−3.14–3.14	−3.14–3.14	−3.14–3.14
Direction index (0;1)	0.55 ± 0.50	0.47 ± 0.50	0.48 ± 0.50	1.00	0.00	0.00	0.00–1.00	0.00–1.00	0.00–1.00
Total distance travelled (km/day)	7.56 ± 6.24	3.96 ± 3.63	3.71 ± 3.06	6.18	3.13	3.04	0.01–44.82	0.00–24.72	0.01–23.35
Net distance travelled (km/day)	4.02 ± 4.67	1.81 ± 2.49	1.53 ± 1.76	2.55	0.91	0.93	0.00–36.27	0.00–21.37	0.00–15.02
Landscape variables									
Dist. to secondary roads (m)	576 ± 900	289 ± 295	146 ± 135	288	195	103	0–11,379	0–2990	0–975
Dist. to railways (m)	24,988 ± 17,065	7135 ± 6171	6710 ± 3245	22,433	4684	6700	0–91,394	0–33,182	0–15,710
Dist. to human habitations (m)	2813 ± 2815	783 ± 654	1049 ± 641	1943	630	947	14–26,819	0–4548	1–3243
Terrain roughness index	18 ± 9	84 ± 40	91 ± 43	16	76	88	0–120	4–328	3–235
Daily median dist. sec. roads (m)	595 ± 907	294 ± 264	146 ± 121	293	214	106	5–11,086	0–2254	0–849
Daily median dist. railways (m)	25,304 ± 17,685	7165 ± 6114	6870 ± 3237	22,439	4717	6877	283–89,019	100–30,696	141–15,436
Daily median dist. h. hab. (m)	2898 ± 2907	817 ± 608	1078 ± 589	1950	684	977	236–23,004	6–4325	18–3243
Daily median terrain roughness index	18 ± 07	87 ± 37	94 ± 39	16	80	94	4–71	6–291	3–216

## Appendix 3 Detailed Linear Mixed Model Outputs for Study Variables

**TABLE A4:** Model coefficients from the best‐fit models obtained for each movement parameter for brown bears. The table shows the exponentiated coefficients obtained from the Linear Mixed effect Models, calculated to transform the logarithmic scale of the speed (km/h), total and net distance travelled (km/day).

Predictors	Speed			Direction index			Daily total distance			Daily net distance		
	exp (β)	exp (CI)	*p*	*β*	CI	*p*	exp (β)	exp (CI)	*p*	exp (β)	exp (CI)	*p*
(obs/2 h or day avg)												
Intercept	0.08	0.06–0.10	**< 0.001**	0.35	0.18–0.51	**< 0.001**	5.08	3.20–8.07	**< 0.001**	2.51	1.23–5.09	**0.011**
RO	0.44	0.39–0.51	**< 0.001**	−0.44	−0.58 to −0.30	**< 0.001**	0.43	0.29–0.64	**< 0.001**	0.21	0.11–0.39	**< 0.001**
SL	0.35	0.31–0.40	**< 0.001**	−0.48	−0.62 to −0.34	**< 0.001**	0.33	0.22–0.49	**< 0.001**	0.19	0.10–0.35	**< 0.001**
scale (DSR)	0.97	0.96–0.98	**< 0.001**	−0.01	−0.02 to 0.00	0.115	0.94	0.92–0.97	**< 0.001**	0.96	0.92–1.00	**0.033**
scale (DRW)	0.99	0.97–1.01	0.186	−0.03	−0.05 to −0.02	**< 0.001**	1.01	0.98–1.05	0.482	0.95	0.90–1.00	0.061
scale (DHH)	1.07	1.06–1.09	**< 0.001**	0.02	0.00–0.04	**0.015**	1.07	1.04–1.10	**< 0.001**	1.1	1.05–1.15	**< 0.001**
scale (TRI)	1.29	1.16–1.44	**< 0.001**	0.14	0.03–0.25	**0.015**	1.19	0.89–1.59	0.25	1.76	1.11–2.79	**0.017**
RO: scale (DSR)	1.07	1.04–1.10	**< 0.001**	0.05	0.02–0.08	**0.002**	1.46	1.37–1.56	**< 0.001**	1.7	1.54–1.89	**< 0.001**
SL: scale (DSR)	0.76	0.72–0.80	**< 0.001**	−0.09	−0.15 to −0.04	**0.001**	0.7	0.62–0.78	**< 0.001**	0.77	0.65–0.93	**0.005**
RO:scale (DRW)	1.25	1.21–1.30	**< 0.001**	0.17	0.13–0.21	**< 0.001**	1.17	1.09–1.26	**< 0.001**	1.6	1.43–1.80	**< 0.001**
SL: scale (DRW)	0.67	0.63–0.71	**< 0.001**	−0.02	−0.08 to 0.04	0.495	0.62	0.56–0.70	**< 0.001**	0.73	0.61–0.88	**0.001**
RO:scale (DHH)	0.76	0.73–0.79	**< 0.001**	−0.11	−0.16 to −0.06	**< 0.001**	0.9	0.81–0.98	**0.022**	0.92	0.79–1.07	0.275
SL: scale (DHH)	0.88	0.85–0.92	**< 0.001**	−0.01	−0.05 to 0.03	0.619	1.13	1.04–1.22	**0.003**	1.27	1.12–1.45	**< 0.001**
RO:scale (TRI)	0.53	0.48–0.59	**< 0.001**	−0.28	−0.39 to −0.17	**< 0.001**	0.59	0.44–0.80	**< 0.001**	0.42	0.26–0.67	**< 0.001**
SL: scale (TRI)	0.49	0.44–0.55	**< 0.001**	−0.25	−0.37 to −0.14	**< 0.001**	0.53	0.40–0.71	**< 0.001**	0.35	0.22–0.56	**< 0.001**
*Random effects*												
*σ* ^2^	3.86			3.29			1.37			3.48		
τ_00 fyear_	0.26			0.05			0.35			0.66		
ICC	0.06			0.01			0.2			0.16		
N _fyear_	21			21			21			21		
Observations	171,960			171,852			16,467			16,439		
Marginal *R* ^2^/Conditional *R* ^2^	0.047/0.106			0.008/0.022			0.120/0.297			0.069/0.217		

*Note:* *P*‐values of statistically significant predictors given in bold.

Abbreviations: DHH, distance to human habitations; DRW, distance to railways; DSR, distance to secondary roads; TRI, terrain roughness index.

**TABLE A5:** Five top models by brown bear movement variable analysed, extracted from the list of all combinations run and ranked based on AIC. All top models include interactions with the predictor variables and study area. The weights of the following models indicate the importance of the interaction terms. When the interactions are removed from any of the predictors, models explanatory power decreases significantly (weight < 0.001).

Intercept	farea	scale (DHH)	scale (DRW)	scale (DSR)	scale (TRI)	farea: scale (DHH)	farea: scale (DRW)	farea: scale (DSR)	farea: scale (TRI)	df	logLik	AICc	Delta	Weight
*y1: log (speed)*														
−2.51	+	0.071	−0.011	−0.033	0.258	+	+	+	+	17	−360,227	720,488	0	1
−2.52	+	0.073	−0.011	−0.035	0.257	+	+	NA	+	15	−360,292	720,614	126.54	3.3E‐28
−2.56	+	0.053	−0.011		0.225	+	+	NA	+	14.00	−360,311	720,649	161.73	7.6E‐36
−2.5	+	0.025	0.005	−0.016	0.252	NA	+	+	+	15	−360,310	720,651	163.17	3.7E‐36
−3.31	+	0.068	−0.005	−0.025	−0.407	+	+	+	NA	15	−360,315	720,660	172.05	4.4E‐38
*y2: Direction index*														
0.35	+	0.02	−0.033	−0.011	0.137	+	+	+	+	16	−118,146	236,323	0	0.99941
0.35	+	NA	−0.026	−0.003	0.134	NA	+	+	+	13	−118,157	236,340	16.813	0.00022
0.35	+	0.007	−0.029	−0.006	0.135	NA	+	+	+	14.00	−118,157	236,341	17.824	0.00014
0.33	+	0.014	−0.033	NA	0.126	+	+	NA	+	13	−118,158	236,341	17.972	0.00013
0.34	+	0.018	−0.033	−0.007	0.132	+	+	NA	+	14	−118,157	236,342	18.728	8.6E‐05

Intercept	farea	scale (med_DHH)	scale (med_DRW)	scale (med_DSR)	scale (med_TRI)	farea: scale (DHH)	farea: scale (DRW)	farea: scale (DSR)	farea: scale (TRI)	df	logLik	AICc	Delta	Weight
*y3: log (Daily total distance)*														
1.63	+	0.066	0.012	−0.06	0.17	+	+	+	+	17	−26016.9	52067.9	0	0.99732
1.62	+	0.071	0.01	−0.062	0.17	NA	+	+	+	15	−26024.9	52079.8	11.842	0.00268
0.86	+	0.062	0.017	−0.053	−0.41	+	+	+	NA	15.00	−26032.9	52095.9	28.009	8.3E‐07
1.61	+	NA	0.036	−0.033	0.147	NA	+	+	+	14	−26038.3	52104.6	36.735	1.1E‐08
0.86	+	0.066	0.015	−0.054	−0.406	NA	+	+	NA	13	−26039.6	52105.3	37.338	7.8E‐09
*y4: log (Daily net distance)*														
0.92	+	0.094	−0.049	−0.044	0.563	+	+	+	+	17	−33,620	67,274	0	0.99707
0.92	+	0.118	−0.058	−0.053	0.565	NA	+	+	+	15	−33627.8	67285.7	11.657	0.00293
−0.36	+	0.089	−0.041	−0.033	−0.402	+	+	+	NA	15.00	−33637.2	67304.4	30.38	2.5E‐07
0.89	+	NA	−0.014	−0.005	0.527	NA	+	+	+	14	−33642.2	67312.4	38.356	4.7E‐09
−0.35	+	0.109	−0.048	−0.041	−0.394	NA	+	+	NA	13	−33643.6	67313.1	39.13	3.2E‐09

## Appendix 4

*Finland and Finnish‐Russian Karelia*

In Finland and Russia, large carnivore populations have been managed by hunting over the past century until today with different practices depending on the country (Ministry of Agriculture and Forestry 2022a). In Finland, until the late 1960s and to some extent even in the 1970s, predators were considered pests and could be killed under any circumstances (Mykrä and Pohja‐Mykrä 2015). This caused a decline in the number of brown bears to about 150 individuals in the Karelian population (Pulliainen 1986; MAF 2022b; Rasmus et al. 2020). In the early 1970s local hunting associations in eastern Finland stopped hunting for several years, leading to a recovery of the population numbers (Kaczensky et al. 2013b). Since then, harvest quotas of bears have varied across time periods and regions according to demography and change of the population (Kojola et al. 2003). In the eastern and central regions, harvest increased strongly during the late 1990s, but pressure decreased when a lower number of bears became evident (Kojola et al. 2003).

The bear population has been increasing for a long time in Finland, with a total of 2250–2400 individuals registered in 2022 (Ministry of Agriculture and Forestry 2022a; Zedrosser and Swenson 2023). The binding management plan established different brown bear management areas (Kojola et al. 2020; Ministry of Agriculture and Forestry in Finland 2022). Hunting is restricted to a specific period of the year in all bear management areas, except in the reindeer husbandry area (RHA), in which there are no control measures. Hunting permits for brown bears and other large carnivore species in the RHA are damage‐ based (See Figure A3). This means that they can be issued without quotas if the conditions set out in the international and national legislation are met (Åhman et al. 2022).

On the other hand, in European Russia, hunting pressure on bears was a major cause of population decrease in the middle and second half of the 19th century due to the high commercial demand for gall bladders and fat from the end of the last century (Vaisfield and Pazhetnov 1992). In the 1950s–1960s, unlimited hunting without control caused a significant population decline, and in 1974, limits set by hunting regulations helped stabilise the population. However, bear hunting has always been very popular in Russia, very often done illegally (Braden 2014).

*Romania*

In Romania, the brown bear population experienced major declines during the first half of the 20th century, followed by early restrictive measures. This management approach differed from the management practices and subsequent population growth observed in other European countries (See Figure A4).

As a result of the alarming decrease of the Romanian brown bear population, bear hunting was restricted at the beginning of the 1950s, setting a season for legal hunting, a harvest quota and the obligation to obtain an individual hunting licence (Chapron et al. 2014). Later, during 40 years of communist centralised game administration, hunting was prohibited (Chapron et al. 2014; Popescu et al. 2016; Pop et al. 2018), which resulted in a significant increase in the numbers of bears in the country. The hunting activity resumed after 1989, causing a decline in the population (Linnell et al. 2002). Since then, hunting in Romania has been managed by local hunting organisations following special forestry and hunting plans. This management regime gave rise to an increase in the amount and feeding periods for wildlife (Jurj et al. 2021; Pop et al. 2012; Micu 1998), resulting in an increase of the number of bears, reaching almost 8000 individuals in 1988. Following this peak, a period of decline was registered from 1989 to 1996 due to poaching, illegal use of poisons and very high legal harvest quotas (Anon. 2005).

In order to harmonise national management objectives with international conventions, brown bears were declared as a protected species in Romania in 1996 (Law 103/1996). However, protective measures to obtain a sustainable control of the brown bear population were not implemented until the 2000s (Cazacu et al. 2014). These measures were motivated by the accession of Romania to the EU in 2007. Additionally, trophy hunting of large carnivores was banned in Romania in 2016 (European Parliament, Mission Report on the Management and protection of the brown bear population, 2023).

Currently, bear hunting continues under strict regulations in Romania with hunting quotas varying annually. These quotas are set by the competent authorities. These numbers have been recently raised by the Ministry of Environment, which requested the derogation of the Habitat Directive, as a measure to prevent human‐bear conflicts and guaranteeing people's safety (LEGE nr. 242 din 23 iulie 2024). At the moment, Romania has the highest densities of brown bears among the European population range, with over 50–60 bears/100 km^2^ of habitat (Jurj et al. 2021).

*Slovakia*

In Slovakia the brown bear population has been exposed to high levels of human hunting in the past, being heavily decimated to 20–60 individuals in the early 1930s (Koreň et al. 2011). This severe decline resulted in a total protection year‐round since 1932 (Koreň et al. 2011; Hell and Slamečka 1999). Followed by a gradual increase in population abundance, bear hunting in Slovakia resumed in the form of limited trophy hunting from 1962 (Janík et al. 1986). Currently, the brown bear is strictly protected in Slovakia and the possession and hunting of a bear is possible only based on the permitted exception of the Ministry of the Interior of the Slovak Republic. Following the hunting moratorium of the last 30 years, numbers have increased to over 1000 bears (Figure A5) with average densities of around 5 bears/100 km^2^ and around 10 bears/100 km^2^ in core areas, although the statistics now differ significantly from expert estimates and official game statistics (Rigg & Adamec, 2007a; State Nature Conservancy 2017: https://www.sopsr.sk/web/?cl=251). The continuous growth of the brown bear population and a lack of effective management practices in Slovakia has prompted an increase in illegal killing (Rigg and Adamec, 2007a). Additionally, controversial measures to prevent conflict have been recently implemented in the country, such as the protocols to avoid negative encounters or solve incidents and brown bear habituation “Manual for protective shooting of brown bears”, dated 1st March 2024 (Manuál pre ochranný odstrel medveďa hnedého (2024). Vestník MŽP SR Ročník XXXII Čiastka 1. https://www.enviroportal.sk/dokument/f/manual‐pre‐ochranny‐odstrel‐medveda‐hnedeho.pdf).

*Legislation Texts*

Decree No. 344/2009 Coll., implementing the Hunting Act. In Slovak: Vyhláška č. 344/2009 Z. z., ktorou sa vykonáva zákon o poľovníctve. Available online in Slovak: https://www.slov‐lex.sk/ezbierky/pravne‐predpisy/SK/ZZ/2009/344/20250401.html Accessed on 27 July 2025.

Law 103/1996: Legea fondului cinegetic şi a protecţiei vânatului nr. 103/1996. Law 103/1996, 1996. Available online in Romanian: https://lege5.ro/Gratuit/ge3demzq/legea‐fondului‐cinegetic‐si‐a‐protectiei‐vanatului‐nr‐103‐1996. Accessed on 12 December 2019.

LEGE nr. 242 din 23 iulie 2024 pentru completarea art. 1 din Legea vânătorii și a protecției fondului cinegetic nr. 407/2006, precum și pentru modificarea și completarea Ordonanței de urgență a Guvernului nr. 81/2021 privind aprobarea metodelor de intervenție imediată pentru prevenirea și combaterea atacurilor exemplarelor de urs brun asupra persoanelor și bunurilor acestora, precum și pentru modificarea și completarea unor acte normative. Available online in Romanian: https://legislatie.just.ro/Public/DetaliiDocument/285830. Accessed on 27 July 2025.
