# Correction to “Anthropogenic Infrastructures Shape Brown Bear Movements in Human‐Modified Landscapes”

**DOI:** 10.1002/ece3.73986

**Published:** 2026-07-06

García‐Sánchez, P. , V. Penteriani , M. del Mar Delgado , et al. 2026. “Anthropogenic Infrastructures Shape Brown Bear Movements in Human‐Modified Landscapes.” Ecology and Evolution 16, no. 3: e72680. 10.1002/ece3.72680.41766738 PMC12946484

In the list of authors of this published paper, there should have been two corresponding authors. Ms. García‐Sánchez (first author) has been added as co‐corresponding author, along with Dr. Vincenzo Penteriani.

The online version of this article has been corrected accordingly.

We apologize for this error.

